# *Legionella* SBT applied directly to respiratory samples as a rapid molecular epidemiological tool

**DOI:** 10.1038/s41598-018-36924-w

**Published:** 2019-01-24

**Authors:** Sara Quero, Noemí Párraga-Niño, Miquel Sabria, Irene Barrabeig, Maria Rosa Sala, Mireia Jané, Lourdes Mateu, Nieves Sopena, Maria Luisa Pedro-Botet, Marian Garcia-Nuñez

**Affiliations:** 1 Infectious Diseases Unit, Fundació Institut d’Investigació Germans Trias i Pujol, Hospital Universitari Germans Trias i Pujol, Badalona, Spain; 2 CIBER de Enfermedades Respiratorias, CIBERES, Madrid, Spain; 3 Universitat Autònoma de Barcelona (UAB), Barcelona, Spain; 4 Vigilància Epidemiològica i Resposta a Emergències de Salut Pública, Agencia de Salut Pública de Catalunya, Barcelona, Spain; 5 Hospital Universitari Parc Taulí, Sabadell, Spain

## Abstract

Legionnaires’ disease (LD) is an atypical pneumonia caused by the inhalation of *Legionella*. The methods used for the diagnosis of LD are direct culture of respiratory samples and urinary antigen detection. However, the sensitivity of culture is low, and the urinary antigen test is specific only for *L*. *pneumophila* sg1. Moreover, as no isolates are obtained, epidemiological studies cannot be performed. The implementation of Nested-sequence-based typing (Nested-SBT) makes it possible to carry out epidemiological studies while also confirming LD, especially in cases caused by non-sg 1. Sixty-two respiratory samples from patients with *Legionella* clinically confirmed by positive urinary antigen tests were cultured and tested by Nested-SBT, following the European Study Group for Legionella Infections (ESGLI) protocol. Only 2/62 (3.2%) respiratory samples were culture-positive. Amplification and sequencing of Nested-SBT genes were successfully performed in 57/62 samples (91.9%). The seven target genes were characterised in 39/57 (68.4%) respiratory samples, and the complete sequence type (ST) was obtained. The *mip* gene was the most frequently amplified and sequenced. Nested-SBT is a useful method for epidemiological studies in culture-negative samples, achieving a 28.7-fold improvement over the results of culture studies and reducing the time needed to obtain molecular epidemiological results.

## Introduction

Legionnaires’ Disease (LD) is an atypical pneumonia caused by the inhalation of aerosols containing *Legionella* and is considered to be responsible for between 2% and 13% of cases of community-acquired pneumonia requiring hospitalisation^1,2^. The clinical manifestations of LD are not specific, and laboratory tests are crucial to confirm the diagnosis of the disease. The methods currently used for LD diagnosis are direct culture of respiratory samples, urinary antigen detection, serology, and direct fluorescent-antibody detection^3^. Approximately half of the 59 species of *Legionellae* have been associated with human disease; however, *Legionella pneumophila* is responsible for more than 90% of cases of LD, with serogroup 1 (of the 16 serogroups identified to date) accounting for almost 85% of cases^4–6^. This disproportionately high number of cases of *L*. *pneumophila* sg 1 is generated, in part, by the widespread use of urine antigen detection for LD diagnosis, a method that is specific for *L*. *pneumophila* sg 1.

The reporting of LD is mandatory in Spain and in other countries. Hence, when a case or outbreak of LD is declared, epidemiological studies must be carried out to find the source in order to be able to disinfect it and thus prevent further cases. Furthermore, under Spanish law, there may be major legal consequences for companies on whose premises an outbreak has occurred. Thus, the priority actions to be undertaken in outbreaks of *Legionella* include establishing the exact source of LD as well as ending the outbreak and determining risk factors in order to prevent future outbreaks. The molecular epidemiological tools currently used in *Legionella* laboratories are pulsed-field gel electrophoresis (PFGE) and sequence-based typing (SBT)^3,7,8^, and recently, Whole Genome Sequencing (WGS) has become increasingly popular^9–11^. These methods have great discriminatory power^8^, but they were designed to be applied after obtaining the isolates from samples. The main drawback of molecular epidemiology studies of *Legionella* resides in the low efficiency of recovery from microbiological cultures, even when the protocol is performed by experienced laboratory staff^12,13^.

The European Working Group for Legionella Infections (EWGLI), now renamed the European Study Group for Legionella Infections (ESGLI), has recently improved and optimised the Nested-SBT protocol^14^ originally developed by Ginevra *et al*.^15^. Nested-SBT is a modification of the SBT method, which can be directly applied to respiratory samples. Several studies have evaluated this method’s efficacy in respiratory samples from which the isolate has previously been obtained in order to corroborate the concordance of the results^15,16^.

The aim of this study was to evaluate the utility of Nested-SBT as a rapid molecular epidemiological tool. For this evaluation, a total of 62 respiratory samples previously diagnosed with LD by urinary antigen testing were studied using microbiological culture and Nested-SBT.

## Results

### Microbiological culture and SBT

Only two out of 62 (3.2%) respiratory samples were culture-positive. The isolates were characterised as *L*. *pneumophila* sg 1 Knoxville ST20 and Knoxville ST146 (Table 1).

**Table 1:** Clinical cases included in the study.

Clinical Case	Epidemiological relationship*	Urinary antigen	Culture	ST	*flaA*	*pilE*	*asd*	*mip*	*mompS*	*proA*	*neuA*
1	Sporadic case (03.2017)	+	−	1177	1	4	3	1	2	1	1
2	Sporadic case (02.2017)	+	−		2	4	18	1	−	1	3
3	Sporadic case (12.2016)	+	−	NA1	4	10	15	3	21	14	9
4	Outbreak 1	+	−		3	10	−	10	11	−	6
5	Outbreak 1	+	−	62	8	10	3	15	18	1	6
6	Sporadic case (12.2016)	+	−	NA2	6	4	15	28	9	14	9
7	Sporadic case (12.2016)	+	−		−	10	15	1	9	4	11
8	Outbreak 2	+	−	92	2	3	18	5	5	1	2
9	Outbreak 2	+	−	62	8	10	3	15	18	1	6
10	Outbreak 3	+	−	92	2	3	18	5	5	1	2
11	Outbreak 3	+	−	NA3	4	7	11	3	11	14	9
12	Outbreak 3	+	+	146	2	10	18	10	2	1	6
13	Sporadic case (10.2016)	+	−		−	−	−	−	−	−	−
14	Outbreak 4	+	−	42	4	7	11	3	11	12	9
15	Outbreak 4	+	−		−	−	−	1	11	−	−
16	Sporadic case (09.2016)	+	−	37	3	4	1	1	14	9	11
17	Sporadic case (09.2016)	+	−	NA4	3	10	14	28	9	14	9
18	Sporadic case (09.2016)	+	−	NA5	2	3	6	10	2	5	9
19	Sporadic case (09.2016)	+	−		−	4	3	1	11	5	1
20	Sporadic case (09.2016)	+	−		4	7	−	3	−	12	9
21	Outbreak 5	+	−		−	10	−	10	11	3	20
22	Outbreak 5	+	−		−	4	1	−	−	9	11
23	Sporadic case (04.2016)	+	−		−	−	−	−	−	−	−
24	Sporadic case (04.2016)	+	−		−	−	−	−	−	−	−
25	Sporadic case (02.2016)	+	−	9	3	10	1	3	14	9	11
26	Outbreak 6	+	−	146	2	10	18	10	2	1	6
27	Outbreak 6	+	−	146	2	10	18	10	2	1	6
28	Sporadic case (01.2016)	+	−		−	−	−	−	−	−	−
29	Sporadic case (12.2015)	+	−	448	2	3	18	10	2	1	6
30	Sporadic case	+	−		4	10	3	28	9	4	−
31	Sporadic case	+	−		2	−	1	13	9	1	6
32	Sporadic case (10.2015)	+	−	17	2	10	3	28	9	4	6
33	Sporadic case (10.2015)	+	+	20	2	3	18	15	2	1	6
34	Sporadic case (10.2015)	+	−	NA6	2	10	3	28	9	4	2
35	Sporadic case (10.2015)	+	−	NA7	2	3	18	28	5	4	2
36	Sporadic case (10.2015)	+	−	17	2	10	3	28	9	4	6
37	Sporadic case (10.2015)	+	−		2	10	3	28	9	−	9
38	Sporadic case (10.2015)	+	−	1581	6	10	2	28	9	14	9
39	Sporadic case (10.2015)	+	−		3	4	1	1	−	9	11
40	Outbreak 7	+	−		2	10	3	28	9	−	6
41	Outbreak 7	+	−	444	2	10	22	10	2	1	6
42	Outbreak 7	+	−		2	−	−	10	2	1	6
43	Outbreak 8	+	−	92	2	3	18	5	5	1	2
44	Outbreak 8	+	−	92	2	3	18	5	5	1	2
45	Sporadic case (09.2015)	+	−	62	8	10	3	15	18	1	6
46	Sporadic case (09.2015)	+	−	1581	6	10	2	28	9	14	9
47	Sporadic case (09.2015)	+	−		2	10	3	5	9	1	−
48	Sporadic case (09.2015)	+	−	372	2	10	3	28	9	4	1
49	Sporadic case (08.2015)	+	−	NA6	2	10	3	28	9	4	2
50	Sporadic case (07.2015)	+	−		2	4	3	28	−	3	11
51	Sporadic case (07.2015)	+	−	NA8	2	4	3	28	9	1	11
52	Sporadic case (07.2015)	+	−	NA9	3	9	2	5	3	41	15
53	Sporadic case (05.2015)	+	−		−	−	−	−	−	−	−
54	Sporadic case (01.2015)	+	−		2	3	3	28	−	1	6
55	Sporadic case (01.2015)	+	−	17	2	10	3	28	9	4	6
56	Sporadic case (01.2015)	+	−	NA10	12	9	26	5	26	4	15
57	Sporadic case (11.2014)	+	−	372	2	10	3	28	9	4	1
58	Sporadic case (11.2014)	+	−		2	7	−	28	9	4	9
59	Sporadic case (11.2014)	+	−	NA11	2	7	3	28	9	4	9
60	Sporadic case (05.2017)	+	−	1	1	4	3	1	1	1	1
61	Sporadic case (05.2017)	+	−		−	4	−	1	−	−	−
62	Sporadic case (05.2017)	+	−	146	2	10	18	10	2	1	6

Urinary antigen and culture results are marked as (+) (positive) or (−) (negative) results. An (−) in the allelic profile indicates a failed gene. NA: no ST assigned in the ESGLI database.*Epidemiological relationship between cases before the molecular results; in sporadic cases the month and year of the sample are included in brackets.

### Nested-SBT

Amplification and sequencing of Nested-SBT genes were successfully performed in 57 out of 62 respiratory samples (91.9%). In 39 samples (68.4%) the seven target genes were characterised and a complete sequence type (ST) was obtained. In 11 samples six genes were characterised, in four samples five genes, in one sample four genes and in two only two genes. In five (8.1%) samples positive by the urine antigen test, none of the seven target genes were amplified (Table 1).

The 39 samples with SBT profiles belonged to 25 different STs (index of diversity [IOD]: 0.972; 95% CI: 0.952–0.991). Eleven STs were new SBT combinations, and 14 were already in the EWGLI database. The most frequent STs were ST92 and ST146, found in four respiratory samples each, followed by ST62 and ST17, found in three respiratory samples each. These four STs represented 28% of the complete STs.

The *mip* gene fragment was the most frequently amplified and sequenced (56 specimens).

Although the ability to obtain typing results did not show concordance (11.3%: kappa index = 0.006, 95% CI: −0.02–0.037) between culture-SBT and direct Nested-SBT, the ST identified in the direct sputum samples was the same as the ST in the *L*. *pneumophila* recovered from the two corresponding culture-positive samples.

## Discussion

Due to the lack of characteristic symptoms of the disease, diagnosis of LD may be difficult. It is therefore vital to have access to accurate diagnostic methods in order to confirm clinical suspicion. The most commonly used method today for the diagnosis of LD is urinary antigen detection together with respiratory sample culture, which is the gold standard^3^. The main drawbacks of urinary antigen detection are that the strains are not isolated to perform molecular epidemiological studies, and that the test is specific only for *L*. *pneumophila* sg 1 and some cases of *L*. *pneumophila* sg 2–15. Indeed, using urinary antigen detection most cases are associated with *L*. *pneumophila* sg 1; around 40% of cases of LD are missed, especially those that are hospital-acquired^6^.

When a case of LD is confirmed, epidemiological studies are carried out in order to identify the source of infection and to initiate disinfection measures so as to avoid the appearance of new cases triggered by the colonised facility. Molecular epidemiological studies are performed by comparing the molecular pattern of the clinical isolates with that of the environmental isolates found in the facilities under suspicion. However, the percentage of recovery of direct respiratory sample cultures is very low^12^.

In the present study, we assessed the utility of the Nested-SBT method as a rapid molecular epidemiological tool. Culture and Nested-SBT were performed in 62 respiratory samples collected from patients previously diagnosed by urinary antigen detection. While the culture of the samples was positive in only two cases, we obtained the complete SBT profile in 39 samples. The two respiratory samples in which the isolates were obtained showed the same ST in both the isolates and the respiratory samples, as reported previously^15–17^. Furthermore, although the SBT profile was not complete, in 18 samples at least two genes were amplified and sequenced.

The previous studies performed using Nested-SBT were based on the determination of the correlation of the results from the SBT of the isolates recovered and from the Nested-SBT of the respiratory samples^15,16^. We assessed the capacity of Nested-SBT to obtain molecular-epidemiological data in culture-negative samples. In culture-SBT, complete sequencing data were obtained in only 3.2% of samples, but in Nested-SBT the figure rose to 91.9% (57/62) – a 28.7-fold increase. These results were similar to those reported by Ginevra *et al*.^15^ and improve the results from studies which applied different semi-nested protocols^12^.

These differences show the great potential of Nested-SBT as a molecular epidemiological tool applied directly in respiratory samples. Although we obtained positive Nested-SBT results in 57 samples, only 68.4% of these were completely typed, obtaining the amplification and sequence of the seven target genes. In the remaining samples only partial amplification and sequencing was achieved; allowing facilities considered as suspicious to be ruled out but not confirming the facility causing the cases or outbreak. Furthermore, the method allowed us to corroborate or rule out the hypothesis of the possible relationship between samples (Table 1). While Nested-SBT confirmed the molecular relationship of cases 26 and 27 and 43 and 44, it ruled out a molecular relationship in cases 4 and 5, 8 and 9, and between 40 and 41 and 42.

The use of *Legionella*-DNA detection methods such as the qPCR described by Mentasi *et al*.^18^ and Benitez and Winchell^19^ avoids the shortcomings of urinary antigen detection and allows identification of the DNA of all the serogroups of *L*. *pneumophila* and all the species of *Legionella*. Furthermore, DNA detection methods are rapid and offer high sensitivity and low cross-reactivity^3,6,20^.

At present, DNA detection is the only method able to diagnose LD due to *L*. *pneumophila* non-sg 1 and *Legionella* non-*pneumophila* within a short time, while the patient is still in hospital and can be adequately treated according to the accurate diagnosis. The sensitivity and specificity of the diagnosis of LD can be increased by combining “classical” and DNA-detection methods, particularly in cases with a low bacterial load and those caused by *Legionella* non-*pneumophila* sg 1, thus allowing identification of cases previously not detected by urinary antigen detection and culture^3^. The cases detected by DNA-method and/or urine antigen can be typed by Nested-SBT, without requiring the isolation of the bacterium. Although Nested-SBT takes from two to five days to obtain the results, its use confirms the diagnosis of LD and also obtains molecular-epidemiological data which can aid epidemiologic studies and shorten the period of six to 10 days needed to isolate the strain and apply the molecular method. Time is decisive in identifying the source of infection and avoiding cases that may result in large-scale outbreaks of LD.

In our study, Nested-SBT failed to detect specific *Legionella* DNA in only 8.1% of LD samples; these failures may have been due to poor sputum quality, or to the administration of antibiotic treatment a few days before sample collection, thus decreasing bacterial load and inhibiting detection. Furthermore, since all the samples were from patients with positive urine antigen, our results show that despite having an incomplete SBT profile, the amplification and sequencing of two genes was sufficient to confirm the diagnosis of cases clinically suspected of LD and to initiate appropriate treatment against *Legionella*.

Assessing the combination of real-time PCR and Nested-SBT to diagnose LD, Qin *et al*.^21^ described a similar efficiency to that recorded in our study. However, they only included samples which were positive with Real-Time PCR; they excluded pneumonia caused by *Haemophilus* and *Streptococcus* and did not assess the specificity of Nested-SBT in order to confirm its utility as a diagnostic tool versus other bacterial pneumonia.

One limitation of Nested-SBT is that the *neuA* gene found in some *L*. *pneumophila* isolates^22^ requires the amplification of the homologous *neuA* (*neuAH*) gene for their characterisation, and the Nested-SBT protocol is not designed to cover the *neuAH* gene. Therefore, cases requiring the *neuAH* gene amplification protocol for sequence typing would only achieve the ST profile of a maximum of six genes, and would always lack the *neuA* gene.

In the present study we confirmed the utility of Nested-SBT for performing molecular epidemiological studies in culture-negative samples. With this methodology we were able to amplify and sequence almost all the cases, while direct culture allowed epidemiological studies in only two. Furthermore, this typing tool reduces the time of typing result from 10–15 days to three to five days, since there is no need to wait for the isolation and growth of the bacteria.

To demonstrate further its utility and power in combination with DNA detection methods (qPCR) for avoiding the culture stage, Nested-SBT should be applied in cases of pneumonia with suspicion of LD in which urinary antigen detection and direct culture are negative, and its specificity should be validated by applying the method to other pneumonias of different aetiological origin.

## Material and Methods

### Sample selection

A total of 62 respiratory samples from patients with *Legionella* clinically confirmed by positive urinary antigen tests from November 2014 to May 2017 were provided by the Epidemiological Surveillance Network of the Catalan Public Health Agency.

The study was conducted in accordance with the Declaration of Helsinki and Spanish law concerning clinical research. The protocols were approved by the local Human Research Ethical Committee at the Germans Trias i Pujol Hospital (Badalona). Since 2001, our laboratory has been the reference laboratory for Legionella typing in LD outbreak/cluster investigations in Catalonia. Since the clinical isolates studied were anonymised and referred to our laboratory for molecular typing by the health authorities in accordance with Spanish legislation, the need for informed consent was waived by the local Human Research Ethical Committee.

### Microbiological culture

The respiratory samples were cultured on buffered charcoal yeast extract (BCYE) agar and glycine vancomycin polymyxin cycloheximide (GVPC) agar or BCYE supplemented with cefamandole, polymyxin B and anisomycin (BMPA) on microbiological agar plates according to the routine microbiological standards in each hospital.

### *L*. *pneumophila* identification and monoclonal antibody (MAb) subtyping

*L*. *pneumophila* isolates were identified by the Monofluo IFA test kit (Genetic Systems Corp., Redmond, WA, USA) and were differentiated as serogroup 1 or non-serogroup 1 by immunoagglutination serotyping (Oxoid Legionella Latex; Germany). The isolates were stored in glycerol-brain heart infusion (BHI) (Oxoid) at −80 °C.

Monoclonal antibodies (MAbs) from the Dresden Panel were used to determine the serogroup of *L*. *pneumophila* non-sg 1 and the phenotypic subgroup of *L*. *pneumophila* sg 1. Serogroup determination was based on the reaction against a specific antibody by indirect immunofluorescence for each serogroup. The phenotypic subgroup of *L*. *pneumophila* sg 1 was based on the reaction against seven MAbs using an indirect immunofluorescence assay, dividing sg 1 into nine different phenotypic subgroups according to a flow chart^23^.

### SBT (DNA amplification from isolates)

Genomic DNA was extracted from isolates obtained from respiratory samples using the Chelex™ extraction technique (Bio-Rad Laboratories, CA, USA). The seven target genes (*flaA*, *pilE*, *asd*, *mip*, *mompS*, *proA* and *neuA*) were amplified using the primers and amplification protocol provided by the ESGLI (V.5)^14^.

### Nested-SBT (DNA amplification from respiratory samples)

The DNA of the respiratory samples was extracted using the Dneasy Blood&Tissue Kit (Qiagen, Germany) following the manufacturer’s instructions. The seven target genes (*flaA*, *pilE*, *asd*, *mip*, *mompS*, *proA* and *neuA*) were amplified using two amplification rounds with the primers and amplification protocol provided by the ESGLI^14^.

We included no template controls (NTC) for each pair of primers in the first and second amplification steps. Furthermore, during the second round of PCR we amplified the NTC of the first round of the nested-PCR. If any NTC was positive, all PCR reactions in that set were considered to be contaminated and were discarded and repeated.

### DNA sequencing and allele assignment

DNA sequencing was performed in a 3130xl (3100) ABIPrism genetic analyser (Applied Biosystems, Foster City, CA) at the Genomics Unit of the Health Science Research Institute Germans Trias i Pujol Foundation. Sequences were analysed using Sequence Scanner software v1.0 (Applied Biosystems). Then, the allele number was assigned using the SBT-ESGLI Quality tool, and the ST profiles were defined with the SBT-ESGLI database.

### Statistical analysis

Descriptive statistics were presented as frequencies with the prevalence (95% confidence interval [CI]) for categorical variables. Concordance between culture and Nested-SBT as a molecular epidemiological tool was analysed using Cohen’s kappa (*k*) test (95% CI). To compare the performance of the diagnostic tests, we applied the McNemar exact statistical test with continuity correction for correlated proportions (p < 0.05).
